# Construction of antibacterial nano-silver embedded bioactive hydrogel to repair infectious skin defects

**DOI:** 10.1186/s40824-022-00281-7

**Published:** 2022-07-25

**Authors:** Qi Dong, Dan Zu, Lingqiang Kong, Sunfang Chen, Jun Yao, Jiawei Lin, Lei Lu, Bing Wu, Bin Fang

**Affiliations:** 1 Department of Spine Surgery, The Central Hospital Affiliated to Shaoxing University, Shaoxing, 312030 China; 2 Central laboratory, The Central Hospital Affiliated to Shaoxing University, Shaoxing, 312030 China

**Keywords:** Silver nanoparticles, Bioderived porcine acellular dermal matrix hydrogel, Infected irregular wounds, Antibacterial and antioxidant properties, Low toxicity

## Abstract

**Background:**

Hydrogels loaded with antimicrobial agents have been widely used for treating infected wound defects. However, hydrogels derived from a porcine dermal extracellular matrix (PADM), containing silver nanoparticles (AgNPs), have not yet been studied. Therefore, we investigated the therapeutic effect of an AgNP-impregnated PADM (AgNP–PADM) hydrogel on the treatment of infected wounds.

**Methods:**

An AgNP–PADM hydrogel was synthesized by embedding AgNPs into a PADM hydrogel. We examined the porosity, moisture retention, degradation, antibacterial properties, cytotoxicity, antioxidant properties, and ability of the PADM and AgNP–PADM hydrogels to treat infected wounds in animals.

**Results:**

The PADM and AgNP–PADM hydrogels were pH sensitive, which made them flow dynamically and solidify under acidic and neutral conditions, respectively. The hydrogels also exhibited porous network structures, satisfactory moisture retention, and slow degradation. Additionally, the AgNP–PADM hydrogel showed a slow and sustained release of AgNPs for at least 7 days without the particle size changing. Thus, the AgNPs exhibited adequate antibacterial ability, negligible toxicity, and antioxidant properties in vitro. Moreover, the AgNP–PADM hydrogel promoted angiogenesis and healed infected skin defects in vivo.

**Conclusions:**

The AgNP–PADM hydrogel is a promising bioderived antibacterial material for clinical application to infected wound dressings.

## Background

For the treatment of nonhealing infected skin wounds, localizing an effective bactericidal dose of drugs such as antibiotics is difficult through intravenous administration because the skin barrier and blood vessels are often destroyed in the wound [1]. Moreover, the multiplication of multidrug-resistant microorganisms increases the difficulty of treating infectious wounds [2]. Current research shows that externally applying antibacterial-drug-loaded composite materials to wounds is an effective treatment method [3]. The ideal externally applied antibacterial material should exhibit long-term resistance to various bacteria, low toxicity, an inflammatory response to improve the blood supply to the infected site, and high moisture retention to provide a good microenvironment for wound healing [4].

Silver nanoparticles (AgNPs) exhibit a broad spectrum of antibacterial effects because they can accumulate in bacterial intima, increase membrane permeability, and interact with sulfur-containing proteins in the bacterial cell wall to rupture the wall [5]. Moreover, AgNPs can enter bacteria and interact with sulfur or phosphorus groups to denature bacterial DNA and proteins. This induces the generation of reactive oxygen species and free radicals through interaction with enzyme mercaptan groups to activate apoptotic pathways [1]. This bactericidal mechanism enables AgNPs to exert antibacterial effects on all bacteria without apparently developing drug resistance. However, AgNPs are toxic to human cells because AgNPs can penetrate cell membranes. AgNP cytotoxicity exhibits a dose-dependent relationship with the NP concentration and is negatively correlated with the nanoparticle diameter [6]. Therefore, AgNPs can be loaded within biomaterials and released slowly to mitigate cytotoxicity [7].

Hydrogels are water-insoluble, crosslinked, fibrous network-structured biomaterials exhibiting high-water content, high porosity, and good biocompatibility. Because hydrogels have previously been applied to sustained-drug-release carriers, they can carry and slowly release AgNPs. Moreover, because hydrogels can moisturize wound surfaces, AgNP-containing antibacterial hydrogel dressings have been developed based on wet-wound-healing theory. To ensure effective antibacterial properties, hydrogel dressings can also provide a good wet and closed environment for wounds; thus, the drugs will not adhere to the wound or cause a secondary avulsion injury [8].

Traditional synthetic hydrogel materials include chitosan, alginate, hyaluronic acid, polyethylene glycol, and mercaptosuccinic acid [9–12]. All these materials are toxic, exhibit poor histocompatibility, are cumbersome to produce, and cause considerable irritation to the skin. In addition, hydrogel synthesis typically requires toxic crosslinking agents such as acrylic acid, polyvinyl alcohol, and glutaraldehyde, which may be cytotoxic and are not easily neutralized/eliminated in vivo [8, 9, 13]. To overcome these limitations, many researchers have developed biological drug carriers [14]. Reportedly, extracellular matrix materials (ECMs) exhibit good biocompatibility, can simulate the original cellular microenvironment, provide chemical and mechanical signals, guide cell adhesion, proliferation, and differentiation, and promote the repair and regeneration of homologous tissues [11, 15]. Qiu et al. observed that hydrogels derived from extracellular bone matrix can promote angiogenesis and osteogenesis [16]. Farnebos et al. demonstrated that tendon-derived ECM hydrogels exhibit specific tendon extracellular matrix components, which promote host cell infiltration and remodeling [17]. Parmaksiz et al. reported that chondrogenic ECM contained cytokines that promote the proliferation and differentiation of chondrocytes and found that bone-marrow mesenchymal stem cells (BMSCs) such as TGF-β1, BMP-7, and IGF were enhanced in chondrogenic ECMs [18]. Thus, cell-free extracellular matrix biomaterials can integrate into regenerative tissues without degrading or requiring removal [19, 20]. Moreover, our previous study demonstrated that the skin extracellular matrix can promote skin healing [14].

**Scheme 1:**
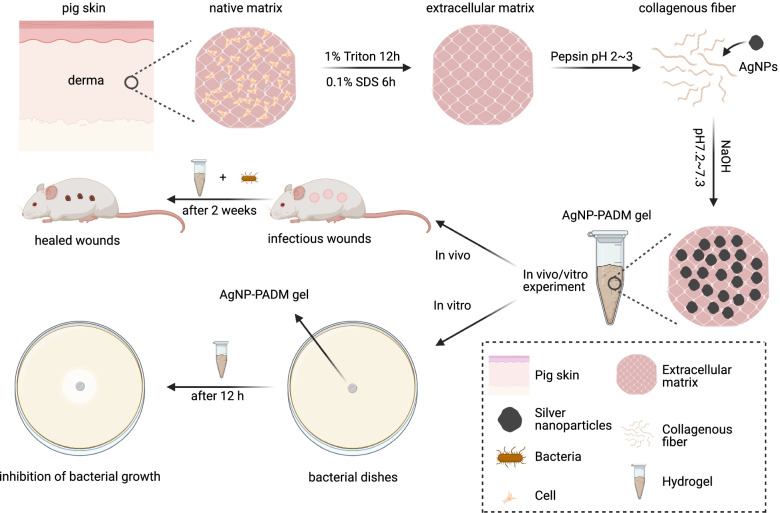
Schematic showing application of the porcine-acellular-dermal-matrix hydrogel-encapsulated AgNPs to heal infectious skin wounds

In this study, a porcine acellular dermal matrix (PADM, a pig-skin extracellular matrix) hydrogel was used as a carrier to encapsulate physically embedded AgNPs to heal infectious skin wounds (Scheme 1). The AgNP–PADM composite hydrogel reduced the AgNP-induced cytotoxicity and transitioned between a liquid and a solid simply by changing the pH, which makes the AgNP–PADM composite hydrogel suitable for application as an antibacterial bandage material to cover various-shaped wounds. The designed AgNP–PADM hydrogel slowly and sustainedly released AgNPs and contributed to long-lasting antibacterial and infectious-wound healing. The porous structure provided a moist microenvironment that promoted epithelial growth within the injured area. The AgNP–PADM composite hydrogel exhibited good antibacterial activity against *Staphylococcus aureus*, *Enterococcus*, and *Enterobacter*. Improving on the PADM hydrogel, the AgNP–PADM hydrogel reduced the inflammatory response of infected wounds and promoted regenerative epithelialization, angiogenesis, and collagen deposition in infected wounds, thereby enhancing wound healability. Therefore, the AgNP–PADM composite hydrogel may be a promising choice for treating infected wounds.

## Methods

### Materials

AgNPs (⌀5 and 50 nm) were purchased from Xi’an Ruixi Biological Technology Co., Ltd. (Xi’an City, China). Triton™ X100, sodium lauryl sulfate and soy peptone were purchased from Sinopharm Chemical Reagent Co., Ltd. (China). Agar, pepsin, and Masson’s trichrome staining reagent were purchased from Solarbio Science and Technology Co., Ltd. (Beijing, China). Tryptone was purchased from OXOID (Shanghai, China), calcein was purchased from Yeasen Biotech Co., Ltd. (Shanghai, China), and Cell Counting Kit-8 (CCK8) was obtained from APExBIO Technology, LLC (USA). Trypsin was purchased from Sigma–Aldrich (USA). The 2,2-diphenyl-1-picrylhydrazyl (DPPH) reagent was purchased from GlpBio (USA).

### Synthesis of AgNP–PADM hydrogel

Pig skin was harvested from fresh adult pig skin tissue collected from local slaughterhouses. The porcine skin tissue was rinsed using sterile water for 3 h and then frozen and thawed three times (between − 80 and 37 °C). The tissue was cut into 1 × 1 cm squares, and the subcutaneous tissue was removed using scissors and shaken at 120 rpm at a constant temperature (25 °C). The subcutaneous tissue was then treated with a 1% Triton™ X-100 solution for 12 h and 0.1% sodium dodecyl sulfate for 6 h. The treated tissue was rinsed extensively in phosphate buffered saline (PBS) and lyophilized. The lyophilized tissue was ground into powder. Then, 20 mg mL^− 1^ of the powder was digested using pepsin in a dilute hydrochloric acid solution (pH 2–3) for 10 min [21], and a ⌀5-nm Ag NP solution was added dropwise, and the mixture was stirred rapidly. Subsequently, this mixture was digested for 2 h until the gel was translucent and viscous and was stored in a refrigerator at 4 °C. PBS solution was added to adjust the osmotic pressure, and precooled 10 M NaOH was added to adjust the pH to 7–8. The gel was maintained at 37 °C for 20 min and was subsequently used to prepare the AgNP–PADM hydrogel.

### Characterization of AgNP–PADM hydrogel

The cellular and nuclear removals were assessed using hematoxylin and eosin (H&E) and 4′6-diamino-2-phenylindole (DAPI) stainings, respectively. The AgNP, PADM hydrogel, and AgNP–PADM hydrogel absorptions were investigated using ultraviolet–visible (UV–Vis) spectrophotometry. The particle size and distribution of the ⌀5-nm AgNPs were observed using transmission electron microscopy (TEM) before the AgNPs were added to the hydrogel and after they were released from the AgNP–PADM hydrogel. The spatial structures of the PADM and AgNP–PADM hydrogels prepared using the 20, 50, and 80 μg mL^− 1^ AgNP solutions were studied using scanning electron microscopy (SEM; JSM-6360LV, JEOL, Tokyo, Japan). After the hydrogels were fixed using 2.5% (w/v) glutaraldehyde for 1 h, the samples were washed thrice with PBS solution and then dehydrated sequentially using 30, 40, 50, 70, 80, 90, 95, and 100% CH_3_CH_2_OH. Subsequently, the samples were dehydrated using liquid carbon dioxide in a critical point dryer and were observed using SEM to structurally characterize the PADM and AgNP–PADM hydrogels. Energy-dispersive spectroscopy (EDS; X-act, OXFORD, England) was used to study the elemental composition and distribution.

The PADM and AgNP–PADM hydrogel porosities were investigated by immersion in isopropyl alcohol and SEM cross-section observation [22]. In the first method, a certain hydrogel volume was prefrozen at − 80 °C for 1 h and then placed in a freeze dryer for 12 h under negative pressure. The solid gel was weighed (*W*_1_), and the hydrogel was then immersed in an isopropyl alcohol solution until saturation. The isopropanol volume before and after hydrogel soaking in the beaker (*V*_1_ and *V*_2_, respectively) was measured. Then, the hydrogel was removed from the isopropanol, excess isopropyl alcohol was gently wiped off the gel surface with gauze, and the soaked hydrogel was reweighed (*W*_2_). The porosity was calculated as follows: Porosity = (*W*_2_ − *W*_1_)/[(*V*_2_ − *V*_1_) *𝛒], where 𝛒 is the isopropanol density. In the second method, the sample cross-section was scanned using SEM, and the hydrogel porosity was calculated using ImageJ software (National Institutes of Health, USA).

### Rheological measurements

Rheological measurements were performed using a rotary rheometer (MCR302). The scanning frequency was set at 1 rad s^− 1^, and the Peltier probes were preheated to 37 °C. The PADM and AgNP–PADM hydrogel acid flow dynamics were adjusted between pH 7 and 8, and the gel was rapidly drawn and filled between two parallel ⌀20-mm plates exhibiting ~ 1-mm clearance. The storage modulus (G′) and loss modulus (G″) of the PADM and AGNP–PADM hydrogels were recorded over time until the fluid gel dynamically solidified.

### Water retention

The same PADM and AgNP–PADM hydrogel masses were incubated at 37 °C, and the sample masses were measured at different time points until the masses stopped changing. The following formula was used to calculate the hydrogel water retention: water retention rate = *W*_2_/*W*_1_ × 100%, where *W*_2_ represents the hydrogel weight measured at each time point, and *W*_1_ represents the initial hydrogel weight.

### In vitro degradation

To measure the hydrogel degradation under different conditions, we placed the PADM and AgNP–PADM hydrogels in a centrifuge tube, incubated them at 25, 37, or 42 °C, and observed the samples regularly to check whether the PADM and AgNP–PADM hydrogels had dissolved. The liquid in the centrifuge tube was promptly aspirated, and the remaining samples were weighed.

In addition, we solidified 500 μL of the PADM and AgNP–PADM hydrogels and placed them in a centrifuge tube containing 1 mL of PBS. Then, 7.5 mg of trypsin was added to the experimental (trypsin) group at 37 °C. When the hydrogels had been immersed for 7 days, both solution groups were refreshed once a day. The PBS and trypsin solutions were removed from the centrifuge tubes at the same time every day, and the hydrogels were rinsed using PBS. The water on the hydrogel surface was wiped off, the sample was weighed, and same solution was readded.

### Release of AgNPs from AgNP–PADM hydrogel

To determine the AgNP release from the AgNP–PADM hydrogel under different conditions, we initially prepared a series of AgNP solutions, measured their UV absorbances, and generated a standardized UV absorbance curve to calibrate the measurements. The AgNP release from the AgNP–PADM hydrogel was then measured at different temperatures. The PADM hydrogels (e.g., 500 μL of the PADM and AgNP–PADM hydrogels) were placed in a centrifuge tube and incubated at 25, 37, or 42 °C for certain times. The dissolved hydrogel solutions were removed for a period, and their optical densities (ODs) were measured at ~ 400 nm. This operation was repeated until the solution OD stopped increasing.

Trypsin is a commercial food-grade enzyme exhibiting increased proteolytic activity and is used to break down collagen [23]. The AgNP release of the PADM and AgNP–PADM hydrogels was also measured using trypsin-containing and pure PBS. A group comprising solid samples prepared using 500 μL of the PADM and AgNP–PADM hydrogels was placed in centrifuge tubes, and trypsin (7.5 mg) and PBS (1 mL) were added. PBS solution (1 mL) was added to another set of centrifuge tubes containing only two samples. Every day, 1 mL of the solution was drawn, and its OD was measured at ~ 400 nm. Then, 1 mL of the corresponding solution was added to the centrifuge tube after the measurement, and this operation was repeated until the removed solution OD stopped increasing.

Finally, we measured the AgNP release from the PADM and AgNP–PADM hydrogels using an ultrasonic crushing instrument. The solid samples prepared using 500 μL of the PADM and AgNP–PADM hydrogels were cut into small pieces and placed in a centrifuge tube. Deionized (DI) water (400 μL) was added, and an ultrasonic shatterer was used to vibrate the samples at 25 and 30 Hz. The samples were vibrated for 30 s and rested for 20 s per cycle to prevent overheating the solutions. After 24 cycles, the samples were centrifuged at 4000 *g* for 3 min, and 200 μL of the supernatant was removed. The OD was measured at ~ 400 nm. The supernatant was added to the sample after the test, and these operations were repeated until the solution OD stopped increasing.

### In vitro antibacterial properties

The AgNP–PADM hydrogel antibacterial potential was investigated in vitro using the Oxford cup method (OCM) and the colony count method (CCM). Gram-negative bacteria (*Escherichia coli*) and gram-positive bacteria (*Staphylococcus aureus* and *Enterococcus faecalis*) were used for the OCM experiments. First, single colonies of *Escherichia coli*, *Enterococcus*, and *Staphylococcus aureus* were added to the medium-containing test tubes, which were shaken at 150 rpm and 37 °C overnight. Logarithmically growing bacteria were selected for the experiment. An appropriate number (0–1.5 × 10^8^ L^− 1^) of bacteria was selected, and the bacterial solution OD was adjusted to 0.1. Then, the number of bacteria was diluted to 10^5^ L^− 1^, and 1/10 of the culture-medium volume was added to a noncoagulated-agar-containing culture medium, which was fully mixed and added to a Petri dish and cooled. Then, the PADM and AgNP–PADM hydrogels were added. The Petri dish was incubated at 37 °C for 12 h, and the bacteriostatic zone diameter around the samples was measured using a scale. Each experiment was repeated thrice.

Subsequently, we selected *Staphylococcus aureus* for CCM detection. First, the PADM and AgNP–PADM hydrogels were cut into 3-mm pieces and sterilized for 2 h under UV light. Then, 2 mL of the *Staphylococcus aureus* (1 × 10^8^ mL^− 1^) solution was added to a test tube and shaken at 150 rpm and 37 °C to culture the bacteria. The bacterial suspension (100 μL) and EP tubes were centrifuged at 2, 4, and 12 h, the supernatant was discarded, and the bacteria were resuspended in normal saline. The bacterial suspension (20 μL) was evenly coated on nutrient agar plates, and the bacteria were cultured for 24 h at 37 °C and then counted.

The hydrogel prepared using the 80 g mL^− 1^ AgNP solution was placed in a centrifuge tube, and was ultrasonically shocked at 25 Hz first for 30 s and then for 20 s. The AgNP release solution (100 μL) obtained at 60, 100, and 140 min was added to an Oxford cup that had been prepared previously as follows. First, an appropriate *Escherichia coli* solution concentration was used, its OD was adjusted to 0.1, and it was diluted to 10^6^ L^− 1^. Then, 1/10 of the culture medium volume was mixed into the nonsolidified agar medium. The culture medium was poured into the Petri dish and cooled, the Oxford cup was placed, and an appropriate volume of the nonsolidified agar medium was readded. After the upper medium solidified, the prepared AgNP release solution was added, and the Oxford cup was removed 12 h later to observe the bacterial removal status at the bottom.

### Cytotoxicity test

The hydrogel (100 μL) was added to a 24-well plate and sterilized using 25 kGy of *γ* radiation. HeLa cervical epithelioid carcinoma cells were seeded at 4 × 10^4^ well^− 1^ in blank Petri dishes and on PADM and AgNP–PADM hydrogels [24]. Dulbecco’s modified eagle medium (DMEM; Hyclone) was added to 10% (v/v) fetal bovine serum (FBS), penicillin (100 μg mL^− 1^), and streptomycin (100 μg mL^− 1^). The cells were cocultured in a humid 5% carbon dioxide environment at 37 °C for 48 h, stained using a calcium lutein staining kit, and observed using fluorescence microscopy at 515 nm.

Cytotoxicity was investigated using CCK8. The PADM and AgNP–PADM hydrogels were immersed in DMEM (hydrogel/medium = 1:5 v/v). The supernatant was collected as a 100% leaching solution and mixed with a particular proportion of DMEM medium to obtain culture solutions containing 0, 20, 50, and 100% leaching solutions. For further experiments, HeLa cells were seeded in 96-well plates at 2000 cells well^− 1^ and were cultured at 37 °C for 24 h. Then, the supernatant medium was removed from each well, and 100 μL of each medium containing different proportions of leaching solution were added to the DMEM medium. The leaching-solution-free DMEM medium was the control. Then, 10 μL of the CCK8 reagent was added to each well on days 1, 3, and 5. Meanwhile, the cells were incubated at 37 °C for 1 h. Absorbance at ~ 450 nm was measured using a spectrophotometer.

### Oxidation resistance

The PADM and AgNP–PADM hydrogel free-radical scavengabilities were measured using the DPPH free-radical scavenging assay [9], in which the ascorbic acid clearance rate was used as the reference standard. Then, the ascorbic acid, the PADM hydrogel, and the AgNP–PADM hydrogel prepared using the 80-μg mL^− 1^ AgNP solution were added to a 0.4 mM DPPH anhydrous ethanol solution, and the solution was stored in the dark for 10 min. An appropriate supernatant volume was obtained from each sample, and its absorbance was measured at 517 nm. The test sample content in the DPPH-containing anhydrous ethanol solution was adjusted until the DPPH anhydrous ethanol solution absorbance corresponding to the AgNP–PADM hydrogel sample stopped changing. The sample free-radical scavengability at a particular concentration was calculated using the formula Scavengability (%) = *C*_2_/*C*_1_*100%, where *C*_1_ represents the difference between the maximum and minimum absorbances of the ascorbic-acid-containing DPPH solution, and *C*_2_ represents the difference between the absorbances of the DPPH solution at this sample concentration and of the pure DPPH solution. The 50% inhibiting concentration (IC_50_) was used to record the median sample concentration required when the oxygen radical clearance rate was 100%.

### In -vivo experiment

Six-month-old rats weighing 200–300 g were purchased from the Zhejiang Academy of Medical Sciences. The rats were reared in separate cages, fed standard feed and tap water, and the cages were maintained at 25 °C and 55% humidity. Day and night were alternated for 12 h. According to the National Institute of Health publication No. 18–23, 1985, which is typically referenced for the care and use of laboratory animals, great care should be taken with rats. The rats were anesthetized using ketamine (30.0 mg kg^− 1^), their dorsal hair was removed, and their skin was rinsed using 70% ethanol. A scalpel and tweezers were used to create three 1-cm^2^ skin wounds on the midline side, 30 μL of a *Staphylococcus aureus* (1 × 10^8^ CFU L^− 1^) solution was dropped into each wound, and the bacteria were given 2 h to fully infiltrate the wounds, which were divided into three groups as follows. Group I was the control group and did not receive any treatment (e.g., naked wound); group II was treated with the PADM hydrogel; group III was treated with the AgNP–PADM hydrogel and covered with gauze, and the PADM and AgNP–PADM hydrogels were refreshed on days 3, 5, 7, 9, and 11.

The wound area reduction (e.g., wound contraction) was used to indicate the therapeutic outcome. The wound contractions were recorded on days 0, 7, and 14. The wound healing area was expressed as a percentage. The wound-shrinkage percentage was estimated using the formula wound-healing ratio (%) = (*C*_1_ − *C*_2_)/*C*_1_ × 100%, where *C*_1_ represents the initial wound area, and *C*_2_ represents the wound area at each measurement time.

On days 7 and 14, half the rats in groups I, II, and III were randomly anesthetized and sacrificed, and the nonwounded skin was biopsied from approximately 5 mm around the wound. The skin tissue was immobilized in buffered formalin (4%) for 2–3 days before tissue processing and paraffin embedding. Tissue sections (5 μm thick) were prepared using a sectioning mechanism and stained with hematoxylin and eosin (H&E). The neovascularization, epidermis, scarring, and granulation tissue were observed and photographed.

Masson’s trichrome staining can be used to dye early and mature collagens in light and dark blues, respectively to assess wound healing. On days 7 and 14, the wound paraffin sections were stained using Masson’s trichrome reagent, and the tissue section collagen content was analyzed under a microscope. Masson’s trichrome stain was designed to distinguish smooth muscle cells from collagen. Additionally, paraffin sections were immunohistochemically stained to measure the CD68 and CD34 expressions in the samples to evaluate the changes in the macrophage and tissue microvessel contents, respectively, during wound healing.

### Statistical analysis

Statistical analysis was performed using GraphPad Prism 8 (GraphPad Software, Inc., USA). The results were expressed as mean ± standard deviation. One- and two-way analysis of variance (ANOVA) were used to determine whether the results were significantly different, and *P* < 0.05 was considered statistically significant.

## Results

### AgNP–PADM hydrogel characterization

**Fig. 1:**
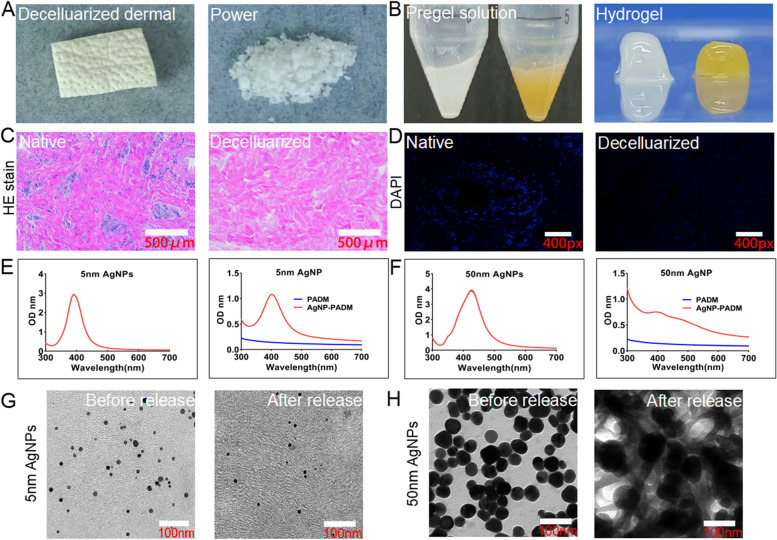
(**A**, **B**) Preparation of AgNP–PADM hydrogels. (**C**, **D**) H&E and DAPI stainings of the porcine skin extracellular matrix showing that cells are completely cleared. (**E**, **F**) 5 nm and 50 nm AgNP solutions and 5 nm AgNP–PADM hydrogels exhibited maximum UV–Vis absorption at 400 nm. 50 nm AgNP–PADM hydrogels did not exhibit any obvious absorption. (**G**, **H**) Changes in 5 and 50 nm AgNP size and diameter before and after AgNPs were released from hydrogel

When the AgNPs were added to the acidic PADM hydrogel solution, the hydrogel color changed from white to pale yellow. After proper agitation and pH adjustment, a hydrogel was formed, and the AgNPs were uniformly distributed (Figs. 1A and B). The H&E and DAPI stainings of the acellular pig skin tissue showed that the pig skin cells had been completely removed and that the pig skin extracellular structure had remained intact, which is consistent with our previous experimental results (Figs. 1C and D) [14]. The UV spectra of the pure 5 nm AgNP solution exhibited a single absorption peak at 400 nm, indicating that the 5 nm AgNPs were uniformly distributed and not aggregated, which is consistent with previously published results [11]. The UV spectra of the liquid AgNP–PADM hydrogels also exhibited a single peak at ~ 400 nm, which confirmed that the 5 nm AgNPs were uniformly distributed in the PADM hydrogels and were not agglomerated (Fig. 1E). We also generated the UV spectra of the 50 nm AgNPs and the corresponding AgNP–PADM hydrogels. Although the 50 nm AgNP UV spectrum exhibited a single peak at ~ 400 nm (Fig. 1F), the 50 nm AgNP–PADM hydrogel spectra did not (Fig. 1F), indicating that the 50 nm AgNPs may have agglomerated in the PADM hydrogel. TEM images revealed that the 5 nm AgNP sizes were negligibly different before the AgNPs had been added to the hydrogels and after the AgNPs were released from the AgNP–PADM hydrogels (Fig. 1G). However, the 50 nm AgNP size significantly increased, P<0.05 (Fig. 1H). The 5 nm AgNP physical properties negligibly changed before and after the reaction, and the 5 nm AgNPs were more uniformly distributed than the 50 nm AgNPs. Moreover, because smaller AgNPs exhibit a larger surface area, they directly contact bacteria more easily and generate superior sterilization [1]. Therefore, the 5 nm AgNPs were selected for the subsequent experiments.

### AgNP–PADM hydrogel fiber structure and porosity

**Fig. 2:**
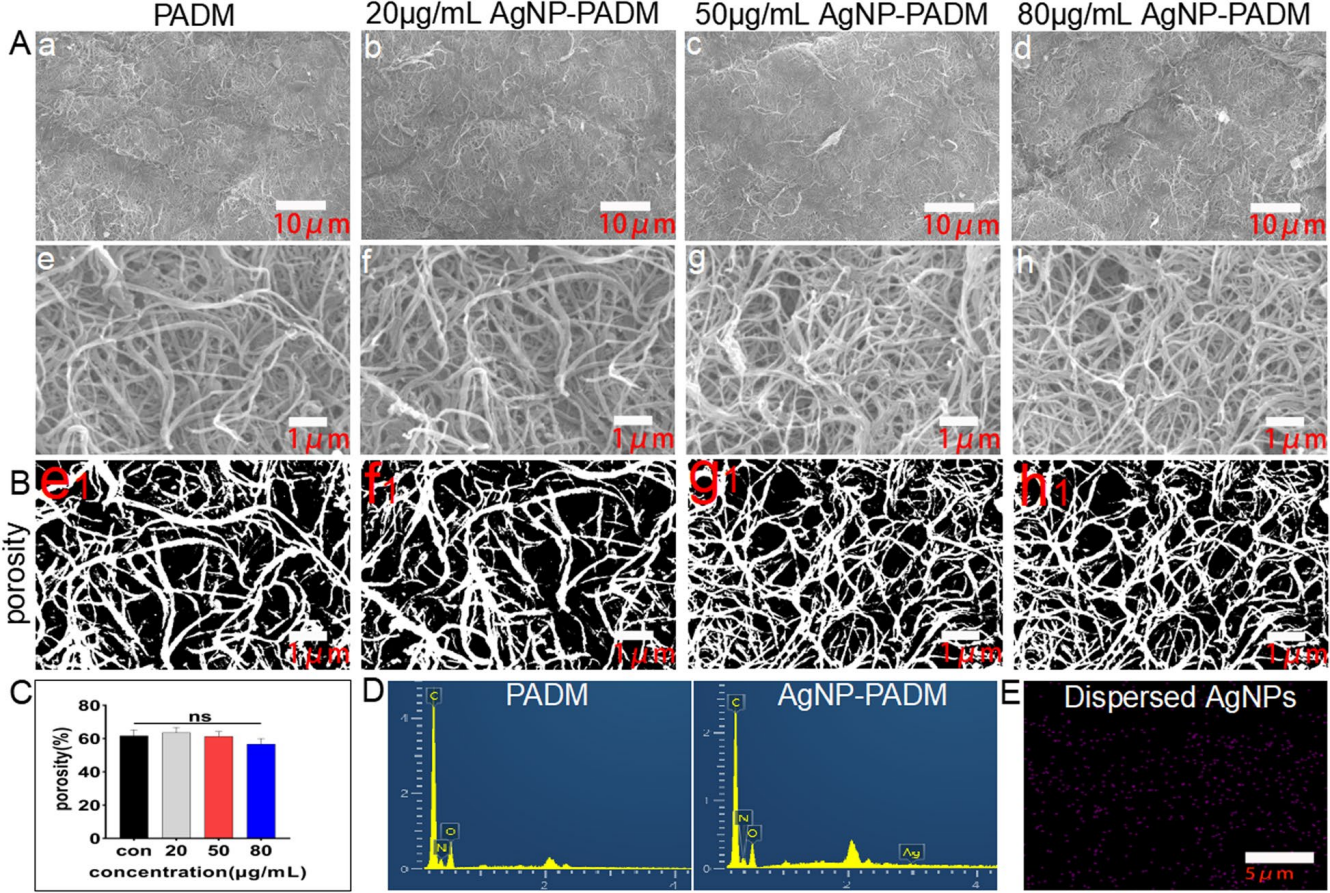
**A** SEM images of PADM hydrogels (PADM) and AgNP–PADM hydrogels (AgNP–PADM) prepared using 20, 50, and 80 μg mL^− 1^ AgNP solutions. PADM and AgNP–PADM hydrogels exhibited three-dimensional reticulated fiber structure. Aa, Ab, Ac, and Ad are magnified by 2000×. Ae, Af, Ag, and Ah are magnified by 15,000×. **B** Porosity calculated using ImageJ software for PADM and AgNP–PADM hydrogels prepared using 20, 50, and 80 μg mL^− 1^ AgNP solutions. Be1, Bf1, Bg1, and Bh1 correspond to Ae, Af, Ag, and Ah, respectively. **C** Porosity of PADM and AgNP–PADM hydrogels prepared using 20, 50, and 80 μg mL^− 1^ AgNP solutions. **D** EDS analysis of PADM and AgNP–PADM hydrogels prepared using 80 μg mL^− 1^ AgNP solution. **E** Element mapping of AgNP–PADM hydrogels prepared using 80 μg mL^− 1^ AgNP solution

The SEM images of the PADM and AgNP–PADM hydrogels are shown in Fig. 2A. The AgNP–PADM hydrogels were prepared using the 20, 50, and 80 μg mL^− 1^ AgNP solutions. Compared with the PADM hydrogel, all the AgNP–PADM hydrogels formed a mesh fiber structure, which is consistent with the results of the extracellular matrix and tendon hydrogels [16, 17], indicating that the pepsin-digested extracellular collagen may reassembled into a collagen-like fiber structure. The PADM and AgNP–PADM hydrogels prepared using the 20, 50, and 80 μg mL^− 1^ AgNP solutions exhibited porosities of 61.67 ± 3.535, 63.57 ± 3.106, 61.30 ± 2.975, and 56.73 ± 3.316%, respectively, *P* > 0.05, indicating that the AgNP concentration negligibly affected the hydrogel porosity (Figs. 2B and C). The EDS maps showed that although the Ag concentration could not be detected in the blank hydrogel group, it was detected in the AgNP–PADM hydrogel prepared using the 80 μg mL^− 1^ AgNP solution) (Fig. 2D). Moreover, the AgNPs were uniformly distributed in the AgNP–PADM hydrogel (Fig. 2E), which is consistent with the corresponding UV absorption spectra and indicates that the PADM hydrogel may be a good AgNP dispersion medium.

### Rheology, water storage, degradation, and release behavior of hydrogels

**Fig. 3:**
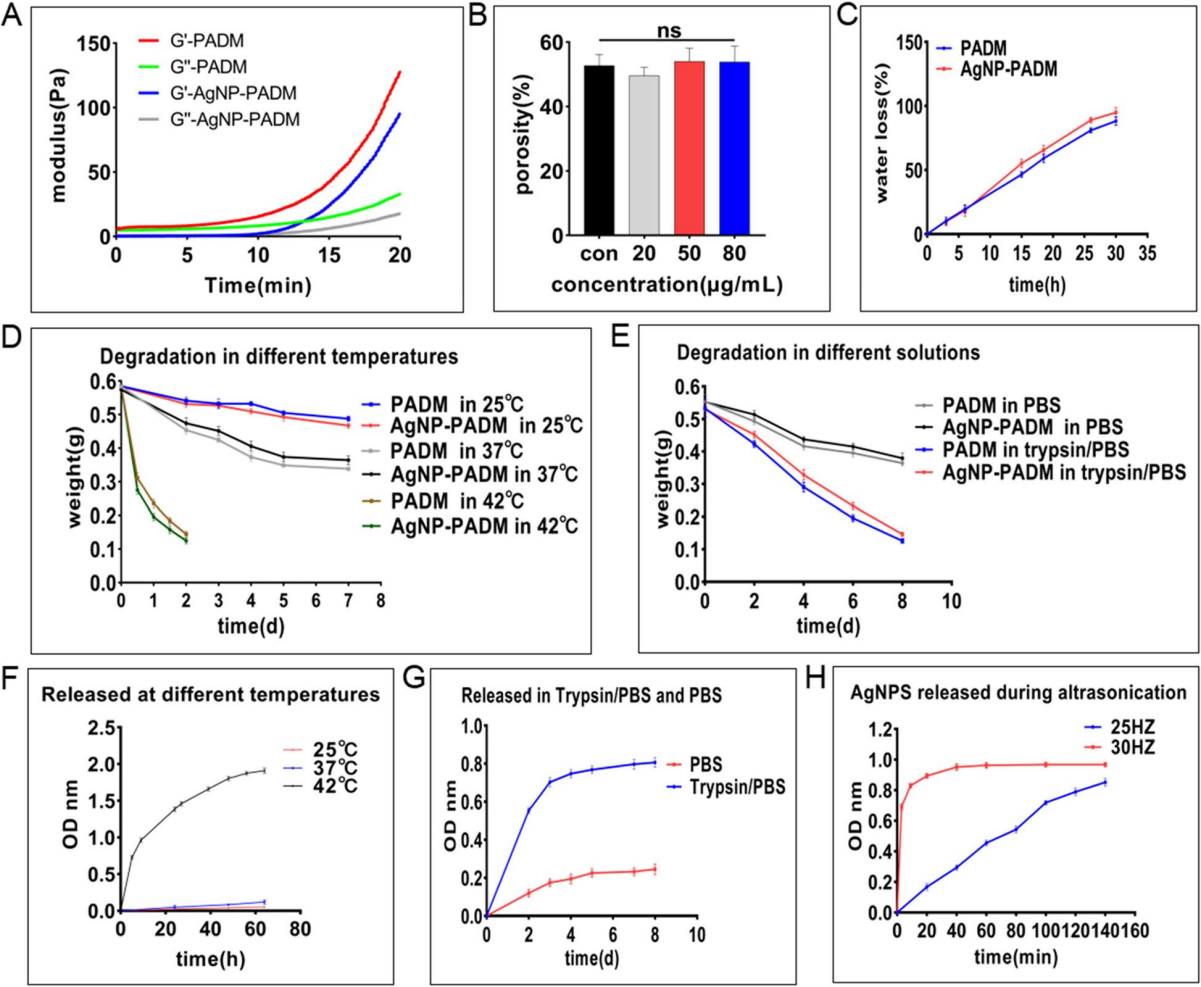
**A** Changes in storage modulus (G′) and damage modulus (G″) of PADM or AgNP–PADM hydrogels over time at neutral pH. **B** Porosity of PADM and AgNP–PADM hydrogels prepared using 20, 50, and 80 μg mL^− 1^ AgNP solutions. All PADM and AgNP–PADM hydrogels exhibited negligibly different porosities. **C** PADM and AgNP–PADM hydrogel prepared using 80 μg mL^− 1^ AgNP solution exhibited negligibly different water retentions. **D** PADM and AgNP–PADM hydrogels prepared using 80 μg mL^− 1^ AgNP solution and subsequently degraded at 25, 37, and 42 °C. **E** PADM and AgNP–PADM hydrogels prepared using 80 μg mL^− 1^ AgNP solution and subsequently degraded with or without trypsin. **F** AgNP release for PADM and AgNP–PADM hydrogels prepared using 80 μg mL^− 1^ AgNP solution and degraded at 25, 37, and 42 °C. **G** AgNP release of PADM and AgNP–PADM hydrogels prepared using 80 μg mL^− 1^ AgNP solutions and subsequently degraded with or without trypsin. **H** AgNP release for PADM and AgNP–PADM hydrogels prepared using 80 μg mL^− 1^ AgNP solution and subsequently ultrasonically oscillated. All values are presented as mean ± standard deviation

The rheological properties of both hydrogels are shown in Fig. 3A. The PADM and AgNP–PADM hydrogels exhibited similar rheological behaviors. For the PADM and AgNP–PADM hydrogels at a neutral pH, the storage (G′) and loss (G″) moduli measured within 5 and 10 min were negligibly different (PADM hydrogel at 5 min, G′: 9.737 ± 5.057 vs. G″: 6.119 ± 3.984, *P* > 0.05; AgNP–PADM hydrogels at 10 min, G′: 6.868 ± 4.274 vs. G″: 5.989 ± 3.731, *P* > 0.05), indicating that the PADM and AgNP–PADM hydrogels were still viscous fluids. With prolonged storage, G′ increased significantly faster than G″, indicating that the gel had gradually transformed into an elastic solid. The AgNP–PADM hydrogel gelatinized slightly slower than the PADM hydrogel, which had completely transformed into an elastic solid within 15–20 minutes. After 20 minutes, the PADM and AgNP–PADM gel G′ and G″ were (PADM hydrogel, G′: 126.3 ± 15.19 vs. G″: 28.99 ± 7.786, *P* < 0.05; AgNP–PADM hydrogel, G′: 98.72 ± 20.29 vs. G″: 22.47 ± 13.39, *P* < 0.05). The porosities of the PADM and AgNP–PADM hydrogels containing different AgNP concentrations were measured using the immersion method. The pristine PADM hydrogel and the hydrogels prepared using the 20, 50, and 80 μg mL^− 1^ AgNP solutions exhibited similar porosities of 52.74 ± 3.406, 49.59 ± 2.559, 54.03 ± 4.069, and 53.84 ± 4.928%, respectively (Fig. 3B). The porosities were statistically insignificant (*P* > 0.05), which is consistent with the results shown in Figs. 2B and C, indicating that a porous network structure can better permeate oxygen, absorb exudate, and provide a cell attachment scaffold, which is also conducive to wound healing [9, 25].

The PADM and AgNP–PADM hydrogel water retentions are shown in Fig. 3C. At 37 °C, both hydrogels lost all their internal water contents after approximately 30 h, and the results were negligibly different for each hydrogel. The AgNPs did not change the PADM hydrogel water storage, which kept the wound wet for a long time, promoted wound healing [8], and guaranteed that a water-based medium was required for uniformly dispersing the AgNPs. Wound defects typically leak fluid, and the hydrogels absorbed part of the seepage while losing water at body temperature, which is also beneficial for wound healing [26].

The PADM and AgNP–PADM hydrogel degradations were carefully measured at different temperatures. As shown in Fig. 3D, the PADM and AgNP–PADM hydrogel degradation rates increased with increasing temperature. Compared with 2 days required to degrade the blank and AgNP–PADM hydrogels to 0.46 and 0.54 g at 25 and 37 °C, respectively, the hydrogels were almost completely degraded in 2 days at 42 °C, and the degradation was significantly accelerated. The hydrogel required more time to degrade at 25 °C, and the degradation increased significantly with increasing temperature.

Trypsin was used to evaluate the PADM and AgNP–PADM hydrogel degradations. The degradation rates at various times are shown in Fig. 3E. On day 4, the PADM and AgNP–PADM hydrogel degradation rates were 24.59 ± 2.755 and 20.67 ± 2.510%, respectively, in PBS. In trypsin, however, the degradation rates were significantly increased (PADM groups: 45.31 ± 2.198 vs. 24.59 ± 2.755%, *P* < 0.05; AgNP–PADM groups: 39.55 ± 1.269 vs. 20.67 ± 2.510%, *P* < 0.05). On day 8, the PADM and AgNP–PADM hydrogel group degradation rates in PBS and pure trypsin were negligibly different (trypsin group: 76.43 ± 0.1044 vs. 72.13 ± 1.227%, *P* > 0.05; PBS group: 34.07 ± 2.416 vs. 31.21 ± 2.829%, *P* > 0.05). The degradation behaviors are similar, which may be because the AgNPs and the PADM hydrogel interact primarily for adsorption, and the AgNPs negligibly affected the enzyme degradation [27]. However, compared with the pure PBS solution group hydrogel degradation rate, the trypsin solution group counterpart was significantly higher, which may be because the decellularized pig skin particles were first decomposed into various collagen fibers during gel preparation and because the PADM hydrogel contained particular trypsin-degradable protein components [28]. These results suggest that during tissue damage, various locally released proteases may degrade other hydrogel protein components and promote AgNP release.

We measured the AgNP–PADM hydrogel AgNP release rates at 25, 37, and 42 °C. As shown in Fig. 3F, the AgNP–PADM hydrogel continuously releases AgNPs for up to 60 h at 42 °C. The AgNPs are released the fastest during the first 10 h, and the release gradually slows until no AgNPs are released at ~ 60 h, which corresponds to complete AgNP–PADM hydrogel dissolution. The AgNP–PADM hydrogel releases AgNPs slower at 37 and 25 °C than at 42 °C. After 1 week, 6.306 ± 0.8149 and 2.835 ± 0.2675% of the total AgNPs are released at 37 and 25 °C, respectively, compared with the amount of AgNPs released at 42 °C, which is consistent with the AgNP–PADM hydrogel degradation rate shown in Fig. 3D.

The AgNP–PADM hydrogel AgNP release behavior was investigated using PBS and trypsin solutions. As shown in Fig. 3G, the trypsin-soaked AgNP–PADM hydrogels exhibit a sustained release of 22.19 ± 0.4058% of the total AgNPs over 8 days. The AgNPs are released significantly slower in the PBS solution than in trypsin one over more than 8 days. The AgNPs are rapidly released on day 3, and then the release slows, and 6.693 ± 0.4163% of the total AgNPs are released on day 8. These results are also consistent with those shown in Fig. 3E.

We also measured the AgNP concentration released from the AgNP–PADM hydrogel by ultrasonic oscillation. As shown in Fig. 3H, the released AgNP concentration increases with increasing oscillation time. Compared with the group ultrasonically shocked at 25 Hz, the group ultrasonically shocked at 30 Hz exhibits significantly accelerated AgNP release, and the maximum AgNP release is measured at approximately 60 min, which is similar to the result obtained for the group ultrasonically shocked at 25 Hz, wherein 10.69 ± 0.1398% of the total AgNP–PADM hydrogel AgNPs are released at approximately 140 min. The AgNP–PADM hydrogel group dissolved at 42 °C releases the highest AgNP content, which is comparable with the maximum AgNP content released in the trypsin and ultrasonic shock groups (Fig. 3H), indicating that some AgNPs still remain in the nondecomposed hydrogels and may be why the internal hydrogel fibrous network structure was dense and the AgNPs were not easily released.

### AgNP–PADM hydrogel bactericidal activity

**Fig. 4:**
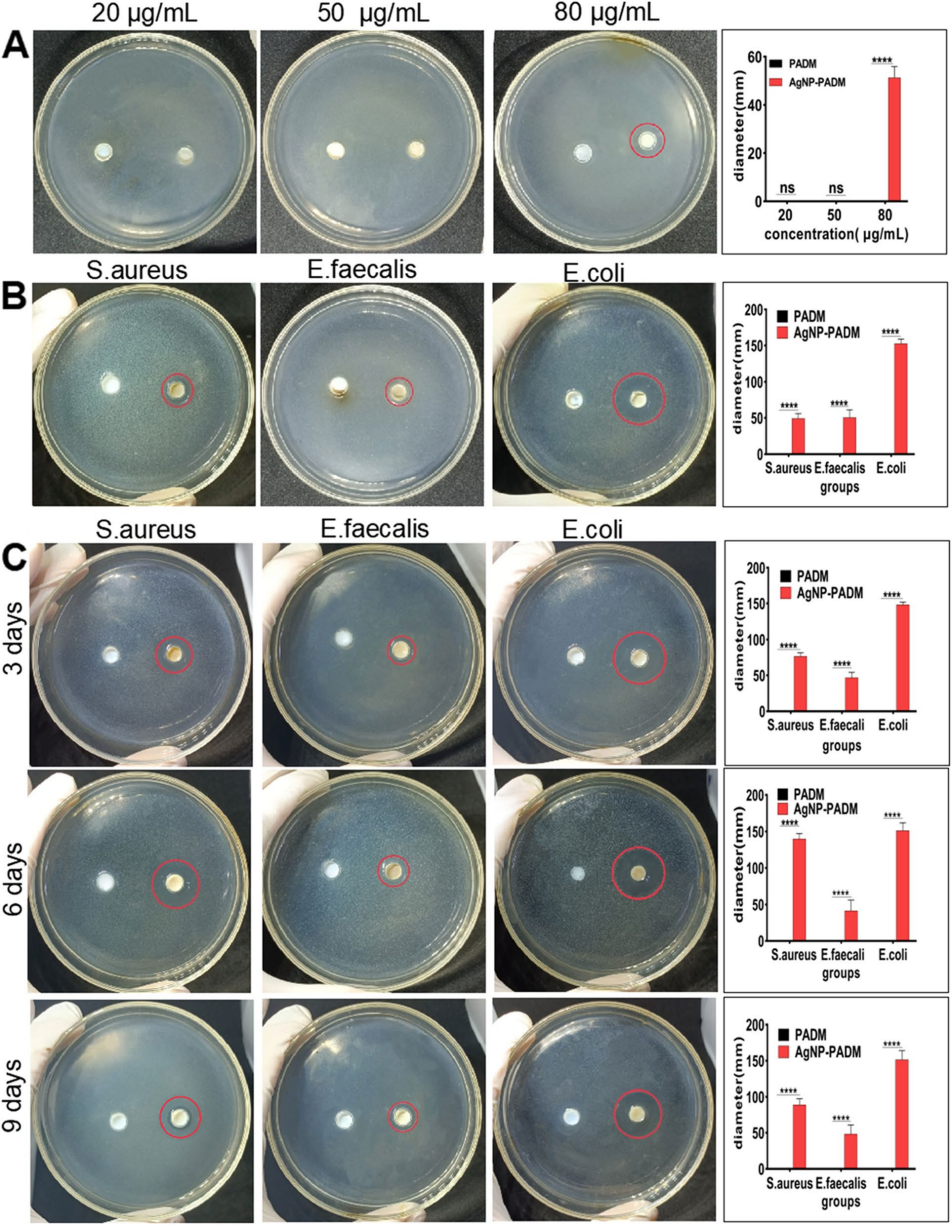
**A** Bactericidal effect of AgNP–PADM hydrogels prepared using 20, 50, and 80 μg mL^− 1^ AgNP solutions on *Enterococcus faecalis*. **B** Bacteriostatic effect of AgNP–PADM hydrogel prepared using 80 μg mL^− 1^ AgNP solution on *Staphylococcus aureus*, *Enterococcus faecalis*, and *Enterococcus Escherichia coli*. **C** Bacteriostatic effects of AgNP–PADM hydrogels prepared using 80 μg mL^− 1^ AgNP solution and stored for 3, 6, or 9 d at 4 °C Bacteriostatic effects of AgNP–PADM hydrogels were negligibly different from those on day one

The PADM and AgNP–PADM hydrogel antibacterial activities were determined using the modified Oxford cup method. The results showed that the antibacterial effects of the hydrogels prepared using the 20 and 50 μg mL^− 1^ AgNP solutions were negligibly different from those of the PADM hydrogel. Because the AgNP–PADM hydrogel prepared using the 80 μg mL^− 1^ AgNP solution exhibited a marked antibacterial effect, we used it for the subsequent experiments **(**Fig. 4A**)**. The diameters of the areas in which the AgNP–PADM hydrogel inhibited the growth of *Staphylococcus aureus*, *Enterococcus faecalis*, and *Escherichia coli* were 49.26 ± 6.852, 50.62 ± 10.65, and 152.8 ± 6.183 mm, respectively, indicating that the AgNP–PADM hydrogels exhibited stronger antibacterial effects than the PADM hydrogels (*P* < 0.05, Fig. 4B). Moreover, the AgNP–PADM hydrogels exhibited stronger antibacterial effects on the gram-negative bacteria than on the gram-positive ones. The AgNP–PADM hydrogels may exhibit less resistance to gram-negative bacterial cell membranes than to the thicker peptidoglycan cell walls of gram-positive bacteria [29]. In addition, we stored the acidic AgNP–PADM hydrogel in a refrigerator at 4 °C for several days and then neutralized the pH to prepare the hydrogels for the inhibition-zone experiment. This effect was negligibly different from the bacteriostatic effect observed on day one (Fig. 4C).

### AgNP–PADM hydrogel planktonic bactericidal effect

**Fig. 5:**
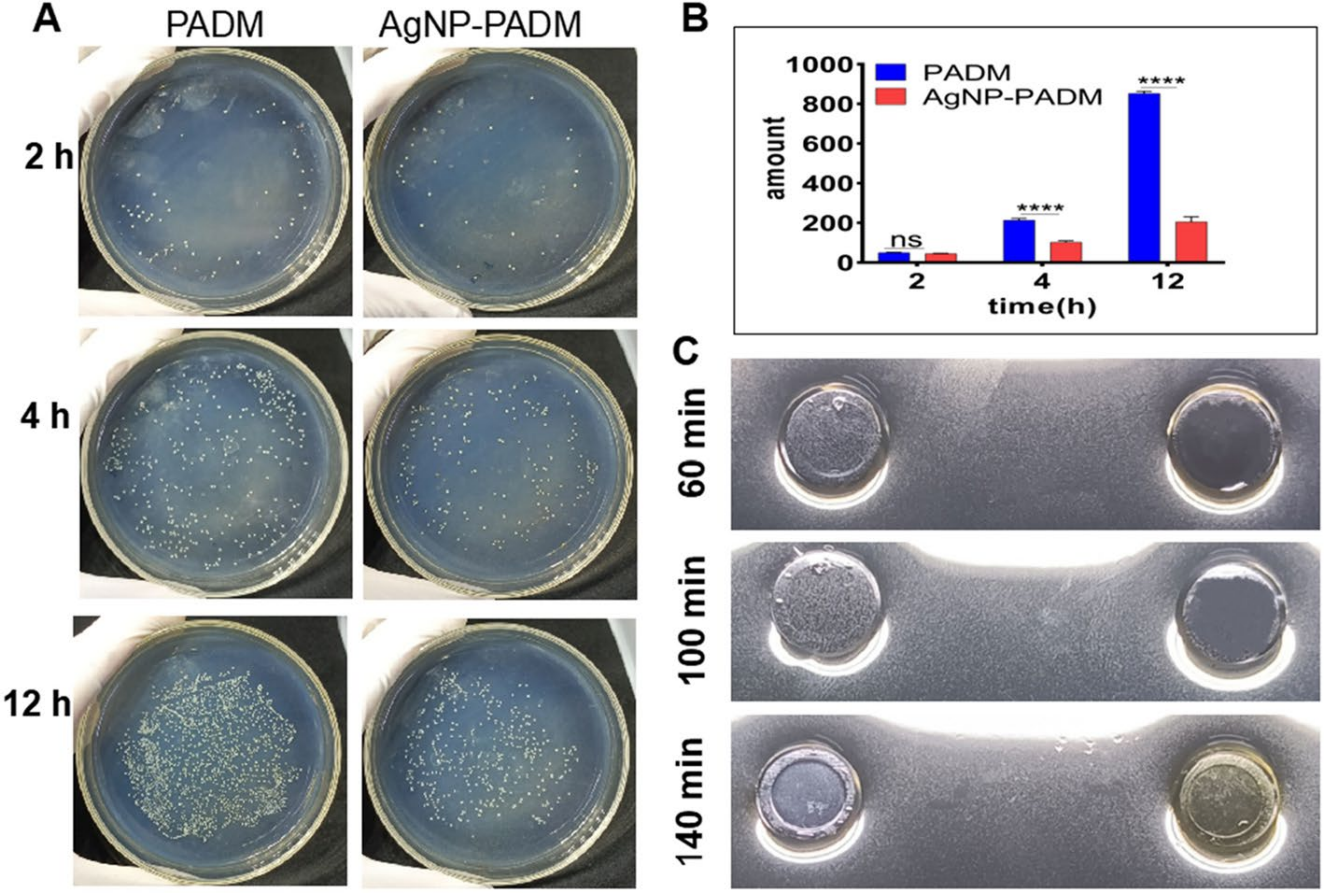
**A**, **B** PADM and AgNP–PADM planktonic bactericidal effect measured using dilution-plate method. **C** Bactericidal effect of AgNP solution released during ultrasonic shocking

The PADM and AgNP–PADM hydrogel planktonic bactericidal effect was investigated using the dilution coating method, as shown in Fig. 5A. Compared with the PADM hydrogel groups, the AgNP–PADM hydrogel groups exhibited lower *Staphylococcus aureus* bacterial counts at 2, 4, and 12 h, and the difference was the most prominent at 12 h. The bacterial counts for the PADM and AgNP–PADM hydrogel groups were 852.7 ± 6.498 and 205.3 ± 14.53, respectively, at 12 h (Fig. 5B), which were consistent with the antibacterial effect of the AgNP–PADM hydrogel in the Oxford cup experiment; that is, the AgNP-impregnated hydrogels exhibited strong antibacterial potential against various bacteria. In addition, we added to the prepared Oxford cup 100 μL of the solutions ultrasonically vibrated at 60, 100, or 140 min to release AgNPs and reperformed the sterilization test. The results showed that AgNPs were released at 60, 100, and 140 min and that all the solutions completely killed *E. coli* in the medium in the wells (Fig. 5C).

### Cytotoxicity, cell proliferation, and antioxidant activity of the AgNP-PADM hydrogel

**Fig. 6:**
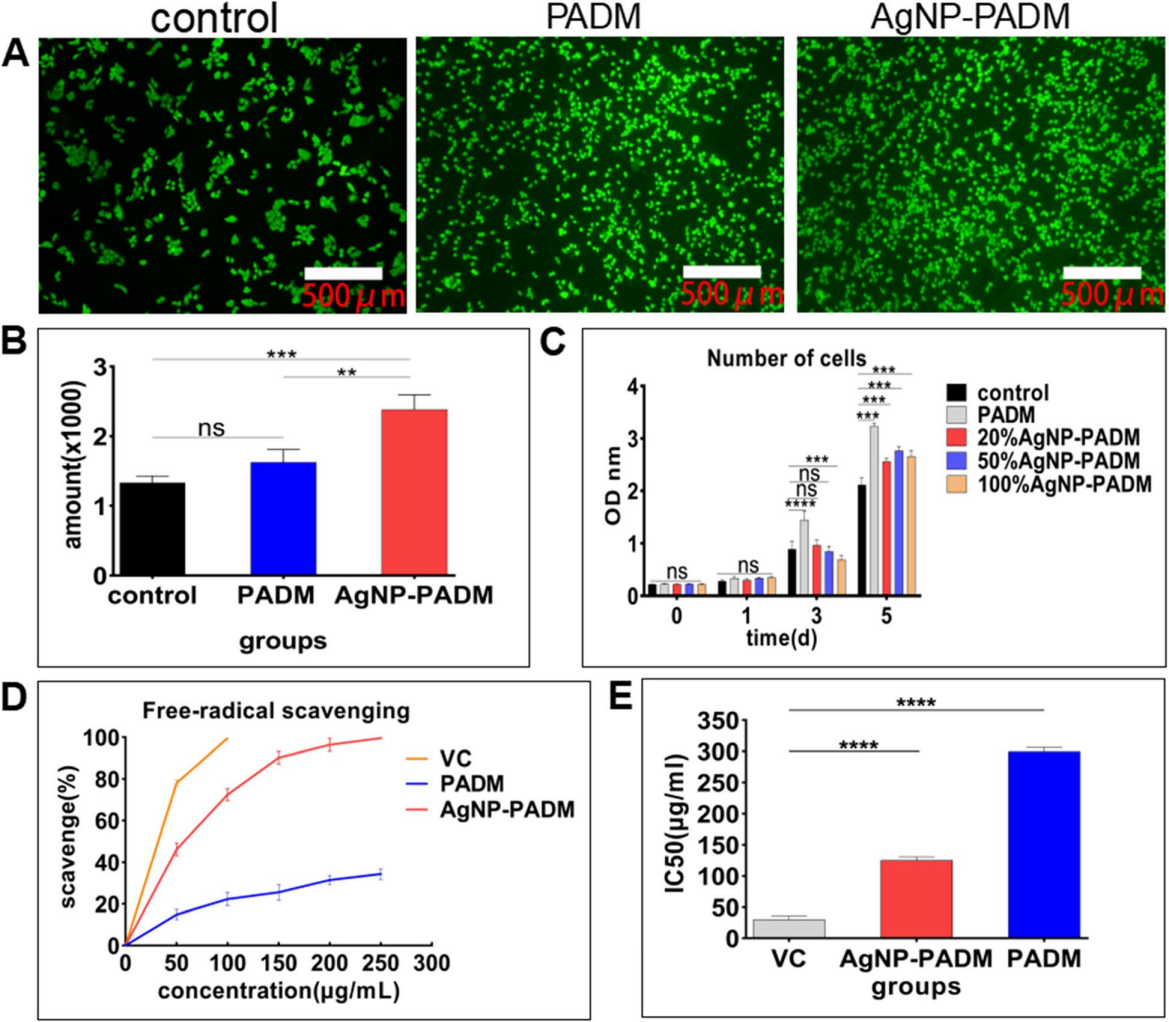
**A**, B Viability of HeLa cells cocultured with PADM and AgNP–PADM hydrogels for 48 h. **C** CCK-8 method was used to detect effects of PADM and AgNP–PADM hydrogel immersion on cell viability. **D** Free-radical scavengabilities of ascorbic acid, PADM, and AgNP–PADM hydrogels in range 50–250 μg mL^− 1^. **E** IC_50_ values of ascorbic acid, PADM, and AgNP–PADM hydrogel groups IC50: the 50% inhibiting concentration

We evaluated the PADM and AgNP–PADM hydrogel cytotoxicities using calcein staining. After the hydrogels were cocultured with HeLa cells for 48 h, the calcein staining results indicated that the number and density of viable cells on the PADM and AgNP–PADM hydrogel surfaces increased significantly compared with those of the viable cells on the blank group surface. Additionally, the cells exhibited good morphology. Thus, although the PADM and AgNP–PADM hydrogels did not produce any detectable HeLa cell cytotoxicity, they did promote cell growth (Fig. 6A and B). In addition, we analyzed the soaking solution cytotoxicity by measuring the PADM and AgNP-PADM hydrogel cellular metabolic activities using the CCK-8 method. As shown in Fig. 6C, both groups exhibit negligibly different cellular metabolic activities on day 1 (*P* > 0.05). On day 3, the number of cells in the PADM hydrogel groups increased significantly, and the number of cells in the 100% AgNP–PADM hydrogel soaking-solution groups decreased compared to the number of cells in the 100% PADM group. On day 5, although the number of cells exhibiting metabolic activities in the PADM and the 25, 50, and 100% AgNP–PADM hydrogel soaking-solution groups increased (*P* > 0.05), the cell survival rate did not decrease. Thus, the PADM and AgNP–PADM hydrogels did not clearly exhibit HeLa cell cytotoxicity, indicating that the AgNP-embedded PADM hydrogel can reduce the AgNP cytotoxicity and that bioderived hydrogels are appropriate biomaterials for growing recipient cells and healing wounds.

The hydrogel antioxidant capacity was measured using the DPPH method, and ascorbic acid was used as the control. The free-radical scavengability of the ascorbic acid and the PADM and AgNP–PADM hydrogels increased in a dose-dependent manner (Fig. 6D). IC50 is the median value of sample concentration required when the scavenging rate of oxygen free radical is 100%. The smaller value of the IC50, the stronger the ability of the sample to scavenge oxygen free radicals will be. In the dose range 50–250 μg mL^− 1^, the ascorbic acid exhibited the highest DPPH free-radical scavengability and the lowest IC_50_ (50 μg mL^− 1^), followed by the AgNP–PADM hydrogel with an IC_50_ of 125 μg mL^− 1^. Furthermore, the PADM hydrogel exhibited partial free-radical scavengability (Figs. 6D and E).

### Repair of infected gap in rats and reduction of local inflammatory response

**Fig. 7:**
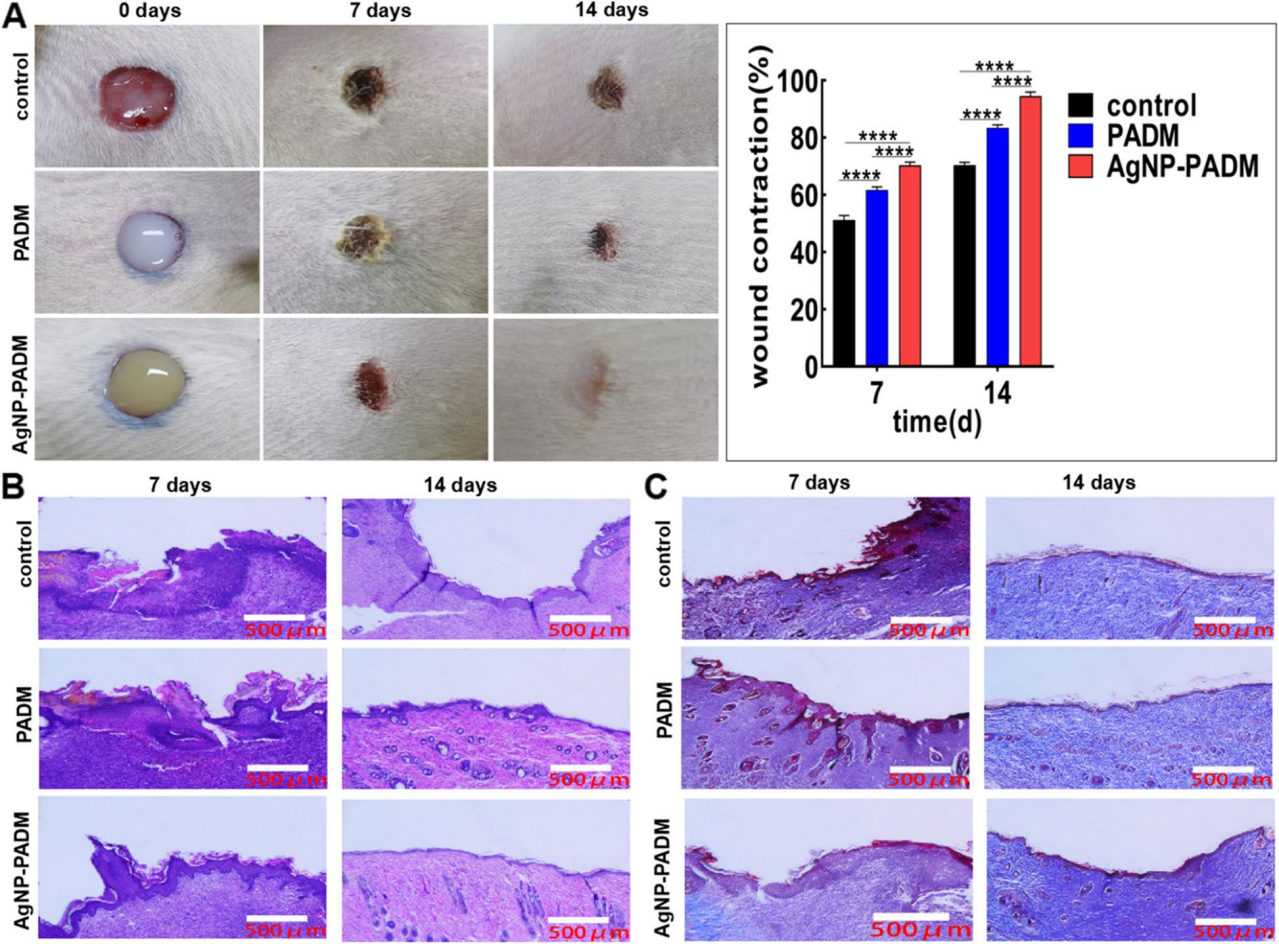
**A** Infected wound healing in PADM and AgNP–PADM hydrogel groups on days 7 and 14. **B** H&E and (**C**) Masson’s staining of wounds the PADM and AgNP–PADM hydrogel groups on days 7 and 14

The experiments demonstrated that the AgNP–PADM hydrogel significantly promoted infected wound healing in rats. After only 7 days, the wounds in the AgNP–PADM hydrogel groups contracted significantly. The wound shrinkage in the AgNP–PADM and PADM hydrogel and control groups were 94.53 ± 0.8090, 83.37 ± 0.6489, and 70.33 ± 0.6064%, respectively. On day 14, the blank and PADM hydrogel group wounds had healed similarly, whereas the AgNP–PADM hydrogel group wounds had healed completely (Fig. 7A).

We used H&E staining to assess wound inflammation and fibroblast development [30], and the results are shown in Fig. 7B. The AgNP–PADM hydrogel groups clearly exhibited continuous epithelial tissue generation and less inflammatory cell infiltration, whereas the blank and PADM hydrogel groups exhibited less epithelial tissue generation and clearly exhibited inflammatory cell infiltration. On day 14, the AgNP–PADM hydrogel group exhibited well-developed hair follicles, whereas the blank group still exhibited a trace of granulation tissue. These results indicate that the AgNP–PADM hydrogel group exhibited accelerated wound healing and repair tissue structures [26].

Collagen deposition was assessed using Masson’s trichrome staining (Fig. 7C). On day 7, slightly more collagen was produced in the AgNP-PADM hydrogel group infected wounds than in the blank and PADM hydrogel group counterparts. On day 14, the collagen production was significantly increased in the infected wounds in all the groups, and the AgNP–PADM hydrogel group exhibited mature collagen deposition and good collagen growth. As an important extracellular matrix component, collagen plays an active role in promoting wound healing [31]. Collagen is formed and crosslinked at the wound site to produce collagen fibers, thereby strengthening the wound tissue.

**Fig. 8:**
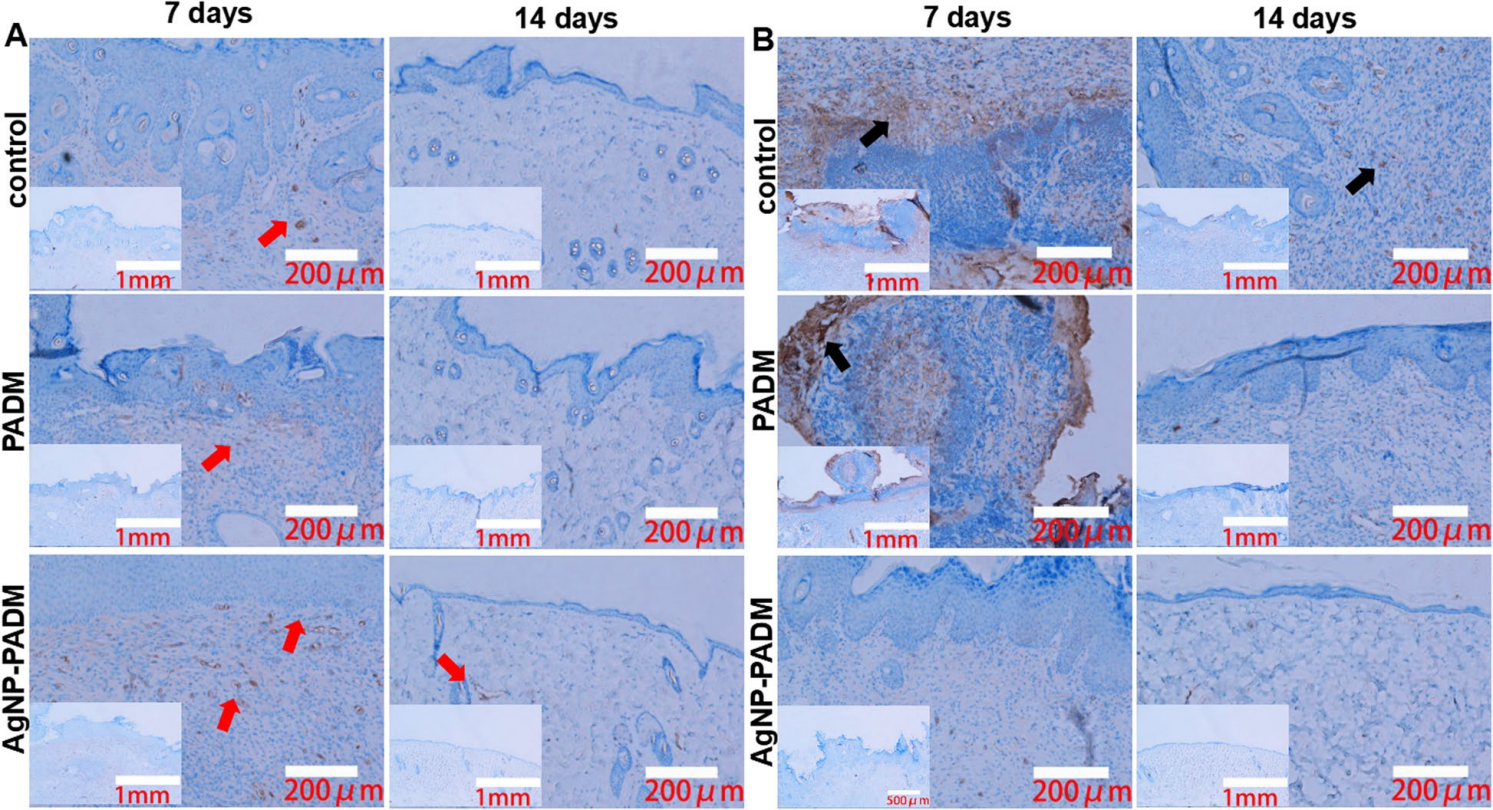
Expression of (**A**) CD34 and (**B**) CD68 in wounds of PADM and AgNP–PADM hydrogel groups on days 7 and 14. Results show that CD34 expression (red arrow) was significantly increased in wounds of AgNP–PADM hydrogel group and that CD68 expression (black arrow) was significantly reduced compared with those of blank and PADM hydrogel groups

We also examined the inflammatory response [32] and angiogenesis [33] during wound repair. The immunohistochemical staining results are shown in Fig. 8. On day 7 after the injury, the CD34 expression was significantly increased in the PADM and AgNP–PADM hydrogel groups than in the blank group (Fig. 8A, red arrow), suggesting that the PADM hydrogel itself promoted wound blood vessel formation, which may be because the PADM hydrogel had maintained its extracellular matrix components [34]. On day 14 after the injury, the CD34 expression in all the groups had decreased compared with those in all the groups on day 7 after the injury, which may be because collagen had gradually accumulated in the wound granulation tissue [35]. On day 7 after the injury, all the groups exhibited different inflammatory response degrees, as measured based on the CD68 expression levels (Fig. 8B**,** black arrow). However, on day 14 after the injury, the CD68 expression level decreased significantly, indicating that the inflammatory response had been alleviated. On days 7 and 14 after the injury, the CD68 expression in the AgNP–PADM hydrogel groups was significantly lower than those in the blank and PADM hydrogel groups. This may be because the hydrogels contained AgNPs, which exhibit specific antiinflammatory effects, reduce the infected wound inflammatory response, and significantly accelerate wound healing. In summary, the AgNP–PADM hydrogel accelerated wound healing by reducing the local inflammatory response, promoting microvascular formation, and accelerating collagen deposition.

## Discussion

We synthesized 5 and 50 nm AgNP-containing PADM hydrogels to determine whether the PADM hydrogels influenced the size and stability of different-diameter AgNPs. Because the AgNP sterilizability negatively correlates with the AgNP size, maintaining the AgNP size and stability is conducive to maintaining effective sterilizability. For example, Rao et al. encapsulated 50 nm AgNPs inside surface-layer proteins through electrostatic interactions and hydrogen bonding to maintain the stability and stagger the release of the AgNPs [36]. Using UV spectra, which are sensitive to the AgNP size [37], we showed that the 50 nm AgNPs had shifted in the PADM hydrogel and had released the solution, whereas the 5 nm AgNPs had remained stable, indicating that the PADM hydrogel had destabilized the 50 nm AgNPs, which might be attributable to the acidic conditions desired during the PADM hydrogel preparation. These conditions destabilized/disintegrated the stable sodium citrate layer, which had previously maintained the uniformly distributed AgNPs [38]. The sodium hydroxide, which was later added, provided numerous hydroxide ions that affected the positively charged silver ions [24]. The AgNPs fused or separated, which changed their size. This result suggests that smaller (e.g., 5 nm) AgNPs are more suitable for loading onto the PADM hydrogel.

Additionally, the acidic AgNP–PADM hydrogel was stored for a week in a refrigerator at 4 °C and still maintained its fluidity, which is consistent with the results of Farnebo et al., who found that the tendon extracellular matrix hydrogel could be preserved under acidic conditions without altering its properties [17]. In addition, the AgNP–PADM hydrogel maintained its antibacterial effect when the pH was neutralized, suggesting that the hydrogel had maintained the homogeneously dispersed 5 nm AgNPs, thereby preserving their potent antimicrobial activity.

Because the AgNP-embedded hydrogels were synthesized in situ by chemical reduction on the hydrogel surface [39, 40], the hydrogels only carried one antibacterial agent. The antimicrobial agents were physically embedded in the PADM hydrogel, which carries not only AgNPs but also other special antimicrobial agents to enhance the hydrogel antibacterial effects [14, 41] by compensating for AgNPs lacking antibacterial ability against certain bacteria.

Additionally, the neutralized AgNP–PADM hydrogel could be plasticized to form different shapes and solidified within 15–20 min, which is beneficial for healing irregularly shaped infected wounds. As the main PADM hydrogel component was biologically derived collagen, the hydrogel was dissolved by pepsin. When the PADM hydrogel was neutralized to the isoelectric points using alkalis, the hydrogel was also reconstituted into collagen fiber networks. For the PADM and AgNP–PADM hydrogels, G′ increased significantly in 20 min. Both hydrogels exhibited increased G′ and G″, indicating that the fluid hydrogels had solidified [42]. The hydrogel recombinant collagen fiber network was susceptible to temperature increases, and high temperatures significantly accelerated hydrogel degradation. Once tissue damage and microenvironmental infection stimulate a local inflammatory response, secondary heat can be released through the bloodstream, thereby increasing the body temperature [43]. Therefore, the accelerated AgNP–PADM hydrogel degradation in infected wounds may contribute to AgNP release, thereby helping to fight the infection. Furthermore, high-temperature environments should be avoided as much as possible in clinical AgNP–PADM hydrogel applications. Although the PADM polymer and collagen triple-helix structures provide stability against hydrogel degradation by enzymes other than collagenase [27], the recombinant collagen fiber degradation could be accelerated by proteases such as matrix metalloproteinases [44], proteolytic enzymes, and collagenases [45, 46]. The release of necrotic cells, inflammation-mediated cells, and fibroblasts during wound healing may somewhat accelerate the AgNP release, which is conducive to maintaining wound sterility [47] and simultaneously decreases the AgNP–PADM hydrogel mechanical strength; therefore, photochemical and chemical crosslinking methods have been developed to slow enzyme-induced hydrogel degradation [48].

ECM hydrogels can maximize the retention of the low-molecular-weight peptides and growth factors in natural ECM [49]. The ECM hydrogel degradation products such as oligopeptide and oligosaccharide derivatives [42] and vascular endothelial growth factors [50] can promote angiogenesis. The CD34 immunohistochemical staining of the rat wound sections suggests that compared to the blank group, the PADM and AgNP–PADM hydrogels significantly increased the intraconal capillary production and accelerated granulation tissue growth, thereby promoting wound healing. Various studies have shown that although homologous-tissue-derived ECM is more conducive to cell growth and promotes the production of the original extracellular matrix components [51–53], the specific components and mechanisms of this phenomenon are not yet fully understood.

The AgNP antibacterial effect, which reduces human keratinocyte viability [54], is usually accompanied by cytotoxicity even when AgNPs are coated with sodium citrate. The significantly increased concentration of AgNP dose-dependent reactive oxygen species is a primary cause of cytotoxicity [55]. Antioxidants can minimize AgNP cytotoxicity [56]. Both PADM and AgNP–PADM were antioxidants, which may be because the collagen-rich PADM and AgNP–PADM hydrogels contained numerous negatively charged amino acids such as proline [23], which can combine with positively charged DPPH to scavenge oxygen free-radicals [9]. It is well known that oxygen free radicals in infected wounds will increase and aggravate local inflammation. Cells in infected wounds release many reactive oxygen species, which can cellularly disrupt the oxidation–antioxidant balance, thereby aggravating tissue damage [57]. The AgNP–PADM hydrogel antioxidant properties are beneficial for reducing the wound inflammatory response and the AgNP cytotoxicity to normal cells, thereby accelerating wound healing [58].

## Conclusions

To treat infected skin defects, AgNP–PADM hydrogels were prepared by embedding AgNPs into a PADM hydrogel, and the hydrogel performance was tested in vivo and in vitro. The AgNPs were uniformly distributed in the PADM hydrogel and slowly released at the body temperature, while maintaining the particle size. The AgNP–PADM hydrogel exhibited significant antibacterial and antioxidant properties, increased nontoxicity, and the ability to promote angiogenesis in vivo. Therefore, the AgNP–PADM hydrogel successfully treated infected skin defects in rats, thereby demonstrating its potential for clinical applications.

## Data Availability

The datasets used and/or analyzed during the current study are available from the corresponding author on reasonable request.

## Abbreviations

AgNPs: silver nanoparticles
PADM: porcine acellular dermal matrix
AgNP–PADM: silver-nanoparticle-impregnated porcine acellular dermal matrix
ECM: extracellular matrix
CCK8: Cell Counting Kit-8
DPPH: 2,2-diphenyl-1-picrylhydrazyl
H&E: hematoxylin and eosin staining
DAPI: 4′6-diamino-2-phenylindole staining
TEM: transmission electron microscopy
SEM: scanning electron microscopy
EDS: energy-dispersive spectroscopy
OD: optical density
OCM: Oxford cup method
CCM: colony count method
